# Comment on: physiology-guided subphenotyping of hypotension in early sepsis

**DOI:** 10.1016/j.aicoj.2026.100043

**Published:** 2026-03-03

**Authors:** Tanmoy Ghatak

**Affiliations:** Sanjay Gandhi Postgraduate Institute of Medical Sciences, Lucknow, Uttar Pradesh, India

Sir,

We read with great interest the editorial by Sanchez and Teboul on physiology-guided subphenotyping of hypotension in early sepsis [1]. The authors should be commended for proposing a pragmatic, bedside-oriented framework that shifts early resuscitation away from uniform protocols toward mechanistically informed, patient-tailored therapy. By emphasizing readily available variables such as capillary refill time (CRT), diastolic arterial pressure (DAP), pulse pressure (PP), and the diastolic shock index (DSI), the editorial offers a clinically intuitive approach to operationalizing personalized resuscitation in routine practice.

Several conceptual and practical considerations, however, warrant further discussion. First, while the proposed hypovolemic, vasoplegic, and cardiac dysfunction subphenotypes are physiologically appealing, sepsis-induced hypotension is rarely driven by a single dominant mechanism. Mixed and rapidly evolving hemodynamic states are common, particularly early in shock, and the relative contributions of hypovolemia, vasodilation, and myocardial dysfunction may change over time [2]. Framing these processes into discrete categories risks oversimplification and may convey a degree of diagnostic certainty that is difficult to achieve using static bedside variables alone.

Second, the reliance on DAP and DSI as surrogate markers of vascular tone, although supported by prior observational studies [3,4], may be confounded in common intensive care unit (ICU) scenarios such as tachyarrhythmias, advanced age with increased arterial stiffness, chronic hypertension, or prior vasopressor exposure. In these settings, a low DAP or elevated DSI may not reliably reflect pure vasoplegia, potentially leading to premature vasopressor escalation or under-recognition of low-flow states. Measurement site and arterial waveform quality may further influence the interpretation of these indices.

Third, although echocardiography is acknowledged as a tool for identifying the cardiac dysfunction sub-phenotype, cardiac ultrasound is positioned relatively late in the proposed diagnostic reasoning. Given the increasing availability of point-of-care echocardiography and its ability to rapidly detect sepsis-related myocardial depression or preload dependence, earlier and more explicit integration of focused cardiac assessment may improve discrimination between hypovolemic and cardiogenic contributors to hypotension.

Finally, while evidence from the ANDROMEDA-SHOCK-2 trial supports CRT-targeted resuscitation in early septic shock [5], it remains uncertain whether a broader physiology-based subphenotyping strategy translates into improved patient-centered outcomes beyond contemporary early vasopressor use and conservative fluid strategies. Prospective validation of this approach, including its reproducibility across operators and settings, will be essential before widespread implementation.

In summary, this editorial represents an important conceptual advance by re-centering early septic shock management on bedside physiology rather than rigid algorithms. Its greatest strength lies in encouraging clinicians to think mechanistically at the bedside. Future studies should focus on validating these subphenotypes in heterogeneous ICU populations, integrating cardiac ultrasound more explicitly, and determining whether this strategy improves outcomes beyond current standard-of-care practices.

## Funding

No funding from anywhere any institution or government.

## Prior abstract publication/presentation

No such presentation in any conference.

## Declaration of competing interest

There is no conflict of interest of any authors involved in this manuscript.
